# New Insights into the Benefits of Physical Activity and Exercise for Aging and Chronic Disease

**DOI:** 10.1155/2017/2503767

**Published:** 2017-10-08

**Authors:** James E. Turner, Vitor A. Lira, Patricia C. Brum

**Affiliations:** ^1^Department for Health, University of Bath, Claverton Down, Bath BA2 7AY, UK; ^2^Department of Health and Human Physiology, Obesity Research and Educational Initiative, Fraternal Order of Eagles Diabetes Research Center, University of Iowa, Iowa City, IA 52242, USA; ^3^School of Physical Education and Sport, University of São Paulo, Av. Prof. Mello de Morais, 65 - Cidade Universitária, 05508-030 São Paulo, SP, Brazil

Diabetes mellitus, cardiovascular disease, and cancer are among the leading causes of morbidity and mortality globally. Strategies to prevent and treat diseases associated with aging and lifestyle have become a priority for health science researchers, guideline groups, and policymakers. The most effective intervention to prevent and, in many cases, treat chronic disease is the adoption of an active lifestyle; however, the cellular and molecular mechanisms by which physical activity and exercise bring about their effects remain poorly understood. This knowledge gap hinders the development of alternative strategies and therapies that could benefit individuals who cannot adhere to structured exercise regimens or make substantial changes to other aspects of their lifestyle. This special issue consists of five original scientific studies and two review articles that investigate or summarise the cellular and molecular processes implicated in chronic disease and their interaction with exercise training. These articles improve our understanding of mechanisms that underpin the preventative or therapeutic effects of interventions for countering disease and raise awareness of current thinking and controversies in aging and lifestyle research.

Two investigations in this special issue focus on cardiomyopathy—hypertrophy and fibrosis of the myocardium that usually causes heart failure—in rat models of obesity (A. C. Silveira et al.) and type I diabetes mellitus (U. Novoa et al.). Exercise is among the most effective interventions for preventing cardiomyopathy. Mechanisms include whole-body systemic processes (e.g., improvements in inflammatory and metabolic profiles) but also exercise-induced alterations that are intrinsic to cardiac tissue. A. C. Silveira et al. demonstrate, in male, five-month old obese Zucker rats exhibiting cardiac hypertrophy, that exercise (60 minutes swimming, +4% body mass applied, 5 times per week, for 10 weeks) increases cardiac expression of microRNA-29c and reduces expression of microRNA-1. Since microRNA-29c targets collagen genes reducing their expression, these findings suggest that exercise may prevent cardiac fibrosis, at least partially, by increasing microRNA-29c levels. In addition, microRNA-1 reduces the expression of the *NCX1* gene encoding a sodium-calcium exchanger, also referred to as solute carrier family 8 member 1 (SLC8A1). Low cardiac NCX1/SLC8A1 expression is common in obesity, and this protein is essential for myocardium relaxation enabling Ca^2+^ release through the sarcolemma. An exercise-dependent reduction of microRNA-1 may improve cardiac function in obesity by restoring gene expression for NCX1/SLC8A1. Thus, microRNA-29c and microRNA-1 might form the basis of new therapies for obesity-associated cardiomyopathies.

In the study by U. Novoa et al., cardiomyopathy was investigated in male, three-month old, Sprague-Dawley rats with type I diabetes induced by Alloxan—a compound with selective toxicity to pancreatic beta cells. Diabetic cardiomyopathy is associated with both type I and type II diabetes independently of confounding factors such as hypertension and coronary artery disease. Multiple cellular and molecular mechanisms have been implicated, including overproduction of reactive oxygen species causing oxidative stress. U. Novoa et al. examined whether high-intensity exercise (10–15 minutes treadmill running, 80% maximum capacity, 5 times per week, for 4 weeks) reversed cardiac remodeling and had the potential to limit cardiac oxidative stress. Exercise training reduced cardiomyocyte hypertrophy and cardiac collagen deposition. However, there was a substantial increase in cardiac apoptosis, which occurred in parallel with uncoupling of endothelial nitric oxide synthase (NOS), and increased gene and protein expression for NADPH oxidase 2 (NOX-2), both potential sources of free radicals and reactive oxygen species. These results suggest that although high-intensity exercise training leads to beneficial effects on cardiac structure, this form of exercise is also associated with cardiac oxidative stress, justifying future studies that examine the effects of different exercise intensities.

The consequences of obesity- and diabetes-associated cardiomyopathy include heart failure with preserved ejection fraction (HFpEF) or reduced ejection fraction (HFrEF). Successful treatments, such as beta blockers, that prolong survival in patients with HFrEF do not improve the prognosis of HFpEF. In this special issue, the review article by A. B. Gevaert et al. advocates a new “whole systems” approach, moving focus from the cardiomyocyte to the endothelium, proposing that exercise interventions could limit or reverse endothelial dysfunction in HFpEF. First, A. B. Gevaert et al. summarise the molecular mechanisms underlying endothelial dysfunction. Second, the characteristics of endothelial dysfunction in HFpEF are described for the vascular beds of different organs, including skeletal muscle, the heart, lungs, and kidneys. Finally, evidence for aerobic exercise training counteracting endothelial dysfunction in HFpEF is presented, with a discussion of possible mechanisms, including increased nitric oxide availability, exercise-induced anti-inflammatory and antioxidative stress effects, and a mobilization of endothelial progenitor cells and angiogenic T cells.

Exercise-induced improvements in nitric oxide availability have clear implications for countering hypertension, but effects beyond the vascular bed also contribute to mitochondrial biogenesis and modulation of bioenergetic pathways in various cell types. Nitric oxide is produced enzymatically by three NOS enzymes: neuronal-NOS (NOS-1), inducible-NOS (NOS-2), and endothelial-NOS (NOS-3). Polymorphisms in NOS genes have been linked to hypertension, NOS enzyme activity, and expression level, but a potential influence on exercise adaptations is poorly understood. In this special issue, A. A. Trapé et al. prescribed multicomponent exercise training to 52 middle-aged overweight and obese women, characterised for three NOS-3 polymorphisms. The 12-week programme consisted of dynamic stretching and strengthening activities plus moderate-to-vigorous aerobic exercise, undertaken for 90 minutes, twice a week. Exercise brought about improvements to various measurements of physical functioning, systolic and diastolic blood pressure, nitrite concentration, and some biomarkers of oxidative stress. However, women within individual NOS3 polymorphism groups exhibited trends towards a smaller magnitude of change for systolic and diastolic blood pressure and nitrite concentration. Women with all three NOS3 polymorphisms did not exhibit exercise-induced improvements in blood pressure or nitrite concentration compared to women without polymorphisms; however, this result should be interpreted with caution due to low sample size. Although confirmation of these results is warranted in larger studies examining different forms of exercise, and the molecular mechanisms remain to be established, some NOS3 polymorphisms might result in impaired exercise-induced enzymatic nitric oxide synthesis. Individuals less responsive to exercise might benefit from dietary supplements rich in nitrate (NO_3_^−^) and/or nitrite (NO_2_^−^), which can lead to nonenzymatic nitric oxide production.

Many studies have examined the effectiveness of interventions for countering obesity-associated changes in adipose tissue mass, inflammation, and metabolic dysfunction. In one contribution to this special edition, D. B. Bartlett et al. examine the utility of a novel composite biomarker—GlycA—for assessing the anti-inflammatory effects of an exercise-based lifestyle intervention. GlycA is a nuclear magnetic resonance spectroscopy signal derived from acute-phase proteins, including *α*1-acid glycoprotein, haptoglobin, and transferrin. GlycA positively correlates with body mass index, markers of metabolic syndrome, and disease activity in auto-immune conditions and is associated with cardiovascular disease and type II diabetes mellitus. D. B. Bartlett et al. randomly assigned 169 overweight or obese men and women with prediabetes, to one of four intervention groups. Three groups undertook different volumes and intensities of exercise prescribed over six months, and one group combined exercise with a calorically restricted diet regimen. On average across the cohort, GlycA concentration decreased significantly by 2% over six months. Slightly larger changes were reported among those undertaking vigorous exercise or moderate exercise plus caloric restriction, but there were no statistically significant differences between groups. Reductions in GlycA occurred in parallel with improvements in body composition and fasting insulin. Thus, GlycA could be a promising biomarker for assessing anti-inflammatory effects of lifestyle interventions, but further work is required to fully understand the influence of exercise dose, plus, or minus diet (e.g., by inclusion of a nonintervention control group).

Overweight and obese individuals seem to exhibit impaired immune function, demonstrated by a greater risk of viral and bacterial infections, smaller immune responses to vaccination, and accumulations of immune cell subsets—some dysfunctional—that are linked to aging. As addressed by two articles in this special issue, exercise training is a potential countermeasure that stimulates immune function, perhaps limiting or delaying the age-associated decline in immune competence, also referred to as immunosenescence. Focusing on innate immunity in a second contribution to this special issue, D. B. Bartlett et al. examined whether different forms of exercise training, in 27 young and middle-aged adults, most classifying as overweight, cause similar immuno-stimulatory effects. Participants were randomised to undertake three supervised group exercise classes each week for 10 weeks, consisting of either high-intensity interval training (18–25 minutes per session, >90% maximum heart rate) or moderate-intensity continuous training (30–45 minutes per session, 70% maximum heart rate). A major finding was that both forms of exercise improved to a similar extent, the capacity of neutrophils to ingest *E. coli* by phagocytosis and subsequently produce reactive oxygen species as part of killing. Thus, by improving innate immune function, both moderate and vigorous exercise training might reduce the risk of bacterial infections in overweight and aging populations. As reviewed by J. Turner and P. Brum in this special issue, the immuno-stimulatory effects of exercise also generalise to the adaptive immune system. J. Turner and P. Brum summarise the mechanisms underlying possible anti-immunosenescence effects of exercise, with a primary focus on the age- and infection-associated accumulation of late-stage differentiated T cells. In addition, studies that have investigated whether immunosenescence influences the risk of developing cancer and affects the treatment of patients with a cancer diagnosis are presented. Finally, it is discussed whether reduced cancer risk and more successful cancer treatment outcomes exhibited by regularly active people are driven by exercise limiting or delaying immunosenescence.

We hope that this special issue is successful in providing new insights into the benefits of physical activity and exercise for aging and chronic disease. As a result, we hope to stimulate new research ideas, investigations, and collaborations, which might, one day, reduce morbidity and mortality from diseases linked to aging and lifestyle.

